# Overexpression of mechanical sensitive miR-337-3p alleviates ectopic ossification in rat tendinopathy model via targeting IRS1 and Nox4 of tendon-derived stem cells

**DOI:** 10.1093/jmcb/mjz030

**Published:** 2019-05-08

**Authors:** Yiyun Geng, Xiaoying Zhao, Jiajia Xu, Xudong Zhang, Guoli Hu, Sai-Chuen Fu, Kerong Dai, Xiaodong Chen, Yung shu-huang Patrick, Xiaoling Zhang

**Affiliations:** 1 Department of Orthopedic Surgery, Xinhua Hospital, School of Medicine, Shanghai Jiao Tong University, Shanghai 200092, China; 2 Shenzhen Key Laboratory of Tissue Engineering, Shenzhen Second People’s Hospital (The First Hospital Affiliated to Shenzhen University), Shenzhen 518035, China; 3 The Key Laboratory of Stem Cell Biology, Shanghai Institutes for Biological Sciences, Chinese Academy of Sciences, Shanghai 200031, China; 4 Department of Orthopaedics and Traumatology, Faculty of Medicine, The Chinese University of Hong Kong, Hong Kong SAR, China

**Keywords:** mechanosensitive miRNA, tendon-derived stem cells, mechanical loading, chondro-osteogenesis, tendinopathy

## Abstract

Tendinopathy, which is characterized by the ectopic ossification of tendon, is a common disease occurring in certain population, such as athletes that suffer from repetitive tendon strains. However, the molecular mechanism underlying the pathogenesis of tendinopathy caused by the overuse of tendon is still lacking. Here, we found that the mechanosensitive miRNA, miR-337-3p, had lower expression under uniaxial cyclical mechanical loading in tendon-derived stem cells (TDSCs) and negatively controlled chondro-osteogenic differentiation of TDSCs. Importantly, downregulation of miR-337-3p expression was also observed in both rat and human calcified tendons, and overexpressing miR-337-3p in patellar tendons of rat tendinopathy model displayed a robust therapeutic efficiency. Mechanistically, we found that the proinflammatory cytokine interleukin-1**β** was the upstream factor of miR-337-3p that bridges the mechanical loading with its downregulation. Furthermore, the target genes of miR-337-3p, NADPH oxidase 4, and insulin receptor substrate 1, activated chondro-osteogenic differentiation of TDSCs through JNK and ERK signaling, respectively. Thus, these findings not only provide novel insight into the molecular mechanisms underlying ectopic ossification in tendinopathy but also highlight the significance of miR-337-3p as a putative therapeutic target for clinic treatment of tendinopathy.

## Introduction

Tendinopathy is a chronic tendon pathology commonly occurring in athletes and population whose tendons suffer from repetitive stretch (Riley, 2008). In addition to tendon soreness, disorganized collagen, vascular ingrowths, and the presence of cartilage and ossified deposits are big challenges to clinical therapy (Riley, 2008; Kongsgaard and Langberg, 2011). Minor injuries caused by repeated strain are considered as the major inducement in tendinopathy, although the mechanism remains unclear (Riley, 2008).

Recent studies have shown that tendon-derived stem cells (TDSCs) reside in tendon tissues of human, mouse, and rat naturally (Bi et al., 2007; Rui et al., 2010) and present common characteristics of stem cells such as clonogenicity, self-renewal capability, and multipotency (Bi et al., 2007). It was previously reported that mechanical loading could induce the differentiation of mesenchymal stem cell from several tissues such as adipose (Tjabringa et al., 2006; Kasper et al., 2007; Kelly and Jacobs, 2010; Ruan et al., 2015). Moreover, osteogenesis of rat tendon-derived stem cells (rTDSCs) under uniaxial mechanical tension (Shi et al., 2012) was previously studied by our group, wherein we demonstrated that osteogenesis of rTDSCs contributes to the ossification in tendinopathy. However, the molecular mechanism underlying this process remains largely unknown.

miRNAs are multifunctional molecules that are broadly involved in many physiological processes, including proliferation, differentiation, apoptosis, and development (Inui et al., 2010; Pasquinelli, 2012; Biggar and Storey, 2018; Han et al., 2018; Sun et al., 2019; Zhu et al., 2019). Previous studies have suggested that mechanical loading can regulate miRNA expression and stem cell differentiation (Guan et al., 2011; Zuo et al., 2015; Yuan et al., 2017). In particular, several miRNAs were reported to be associated with tendon disease. For example, miR-29a was shown to regulate tendon remodeling by targeting IL-33 (Millar et al., 2015). miR-29b-3p could negatively regulate tendongenesis through TGF-β1 signaling (Lu et al., 2017). miR-210 accelerates rat Achilles tendon healing (Usman et al., 2015). Although these results highlight the significance of miRNAs in the regulation of tendongenesis as well as tendon diseases, none of them investigated the roles of miRNAs in the pathogenesis of heterotopic chondro-ossification of tendinopathy. Meanwhile, how mechanical loading influences the chondro-osteogenesis of TDSCs through regulating miRNAs was not reported yet.

In this study, to investigate the molecular mechanisms governing chondro-ossification in tendinopathy, we established a mechanical loading model to induce chondro-osteogenic differentiation of TDSCs *in vitro* and further explored the mechanosensitive miRNAs in TDSCs by miRNA microarray analysis. We found that the expression miR-337-3p was decreased in TDSCs under cyclic mechanical stretch. Furthermore, functional analysis revealed that miR-337-3p plays a negative role in chondro-osteogenic differentiation of TDSCs, and overexpressing miR-337-3p in rat patellar tendinopathy model *in vivo* exerted marked treatment effects. While the mechanical stress was considered as the primary cause that leads to tendinopathy, the molecular mediator has been debated (Aspenberg, 2007; Marsolais et al., 2007; Berkoff et al., 2016). As excessive inflammation was frequently observed in overloaded tendon (Spiesz et al., 2015; Thorpe et al., 2015), we reasoned that inflammatory mediators could be potential candidates for miR-337-3p-mediated tendinopathy. Indeed, we confirmed that proinflammatory cytokine interleukin-1β (IL-1β), but not TNF-α, was activated under mechanical loading in TDSCs, leading to the downregulation of miR-337-3p and subsequent chondro-osteogenic differentiation of TDSCs. Finally, we proved that miR-337-3p modulates chondro-osteogenic differentiation of TDSCs by targeting Nox4–JNK signaling and IRS1–ERK signaling.

## Results

### miR-337-3p plays a negative role in chondro-osteogenic differentiation of TDSCs under mechanical loading

**Figure 1:**
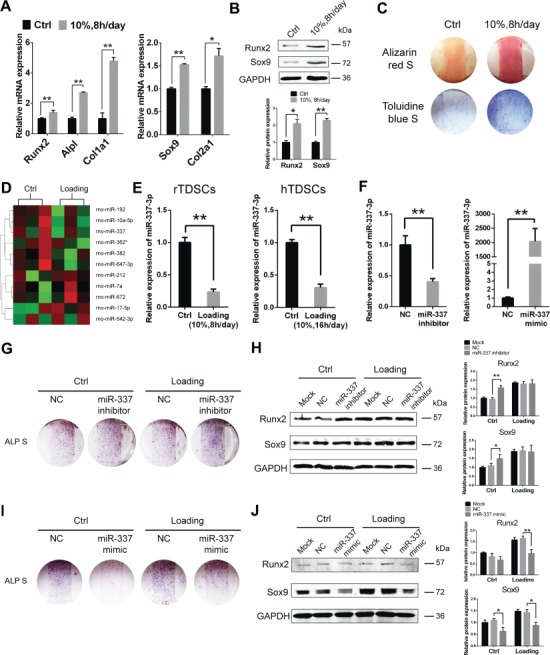
Mechanical loading promotes chondro-osteogenesis of TDSCs through downregulating miR-337-3p. (**A**) Real-time PCR analysis of osteogenic genes (left) and chondrogenic genes (right) in rTDSCs with or without mechanical loading (10%, 8 h/day) on Day 3. Ctrl stands for the non-loading group. *Gapdh* expression was used as an internal control for mRNA expression. (**B**) Western blot analysis of chondro-osteogenic genes in rTDSCs with or without mechanical loading (10%, 8 h/day) on Day 3. The densitometric analysis of Sox9 and Runx2 protein expression was normalized to GAPDH. Three independent experiments were analyzed for the bar graph below. (**C**) Alizarin red staining (upper) and Toluidine blue staining (lower) of rTDSCs cultured for 14 days. (**D**) miRNA microarray analysis of rTDSCs in normal culture and under mechanical loading (10%, 8 h/day) for 7 days. Three samples in each group were analyzed. Eleven differentially expressed miRNAs after normalized analysis are presented in the heat map. (**E**) Real-time PCR analysis of miR-337-3p expression under mechanical loading in rTDSCs (left) and hTDSCs (right) on Day 7. (**F**) Real-time PCR analysis of miR-337-3p expression in rTDSCs transfected with miR-337-3p inhibitor (left) or miR-337-3p mimic (right) on Day 2. (**G**–**J**) Alkaline phosphatase staining of rTDSCs treated with siRNA to inhibit rno-miR-337-3p (**G**) or rno-miR-337 mimics (**I**) along with mechanical loading (10%, 8 h/day) or not for 7 days. Protein levels of Runx2 and Sox9 of rTDSCs in rno-miR-337-3p inhibitor (**H**) or rno-miR-337 mimics treatment (**J**) combined with mechanical loading or not for 3 days. NC stands for negative control siRNA. Three independent experiments of western blot were analyzed for the bar graphs on the right. Mock stands for only transfection reagents-treated group. Error bars, SEM (*n* = 3). *P < 0.05; **P < 0.01.

To choose the proper loading regimen under which TDSCs go into chondro-osteogenetic differentiation, we tested different loading parameters. Under the loading regimen, of which the elongation was 10%, chondro-osteogenic differentiation of both rTDSCs and hTDSCs (Supplementary Figure S1B and C) was notably enhanced. As shown in Figure 1A and B, both RNA (Figure 1A) and protein expression (Figure 1B) of chondro-osteogenic genes, including osteogenic markers like Runt-related transcription factor 2 (*Runx2*), alkaline phosphatase (*Alpl*), and collagen type I α1 chain (*Col1a1*) and chondrogenic markers such as *Sox9* and collagen type II α1 chain (*Col2a1*), were significantly enhanced in rTDSCs after mechanical loading for 3 days. These results were further confirmed by Alizarin Red S staining and Toluidine blue staining on Day 14 (Figure 1C; Supplementary Figure S1D).

To screen for miRNAs involving in the mechanical stretch-mediated chondro-osteogenic differentiation, we compared the expression of miRNAs of cyclic mechanical stretch group and the negative control group in rTDSCs through microarray analysis. A number of miRNAs changed their expression in response to mechanical loading (Figure 1D). Among them, decrease of miR-337-3p under mechanical loading was validated in both rTDSCs and hTDSCs by real-time polymerase chain reaction (PCR) analysis and thereby was chosen for further study (Figure 1E).

To further dissect the function of miR-337-3p in rTDSCs differentiation under mechanical loading, we transfected rTDSCs with either miR-337-3p inhibitor or miR-337-3p mimic (Figure 1F). ALP staining (Figure 1G) and protein change of chondro-osteogenic genes (Figure 1H) showed that miR-337-3p deficiency indeed induced chondro-osteogenic differentiation of rTDSCs. On the other hand, after miR-337-3p mimic transfected, chondro-osteogenic markers were significantly decreased in rTDSCs (Figure 1I and J). From the above, we concluded that miR-337-3p is a mechanosensitive miRNA that functions as a negative regulator in chondro-osteogenic differentiation of TDSCs under mechanical loading.

### Overexpression of miR-337-3p rescues ectopic ossification in rat tendinopathy model

**Figure 2:**
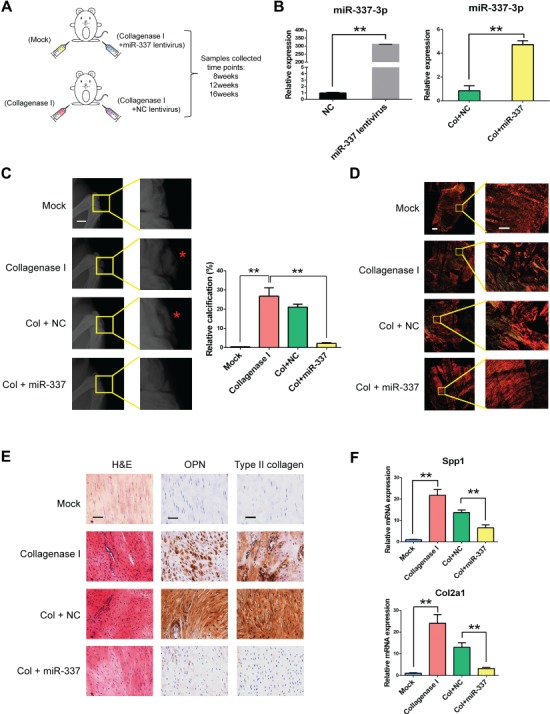
miR-337-3p counteracts chondro-osteogenesis in collagenase I-induced rat tendinopathy model. (**A**) Schematic diagram of applying rno-miR-337-3p overexpressing lentivirus to cure rat tendinopathy induced by collagenase I. All reagents were injected at patellar tendon. Male SD rats (8-week-old) were used for the experiment, and patellar tendon samples were collected after 8, 12, and 16 weeks. Mock group stands for saline injection and suture. (**B**) Real-time PCR analysis of miR-337-3p in rTDSCs transfected with miR-337-3p overexpressing lentivirus for 7 days (left) and in patellar tendon tissues of rat tendinopathy model treated with miR-337-3p overexpressing lentivirus (right). (**C**) X-ray image of the knees of SD rats at 12 weeks after treatment showed that knee joints and ectopic ossicles (red asterisks in the magnified pictures) formed in patellar tendons (left). Scale bar, 5 mm. Relative calcification percents were measured through X-ray results by Image J (right). (**D**) Patellar tendon paraffin sections collected from each group at 12 weeks were treated with Sirius red staining and observed under polarized light microscopy to observe the collagen fibers. Left columns are images of integrated intact patellar tendon. Right columns are enlarged partial patellar tendon images. Scale bar, 800 μm (left) and 200 μm (right). (**E**) H&E staining and immunocytochemistry staining of OPN and type II collagen in each group. Samples were collected at 12 weeks after surgery. Scale bar, 50 μm. (**F**) Real-time PCR analysis of *Spp1* and *Col2a1* in patellar tendon tissues of each group. Error bars, SEM (*n* = 3). **P < 0.01.

Above results has shown that miR-337-3p negatively regulated chondro-osteogenic differentiation of rTDSCs *in vitro*. To further test whether miR-337-3p could be a potential therapeutic target for tendinopathy, we adopted the rat model of tendinopathy induced by collagenase I (Lui et al., 2009). In brief, 3 days after collagenase I injected at patellar tendon, rno-miR-337-3p overexpressing lentivirus as well as control lentivirus were injected at the same position. The effectiveness of miR-337-3p overexpression was confirmed in both rTDSCs and tendon tissues treated with miR-337-3p overexpressing lentivirus (Figure 2B). Then the patellar tendon samples were collected at three time points of 8, 12, and 16 weeks for phenotype analysis (Figure 2A). Surprisingly, the calcification degree of collagenase I-treated group was significantly rescued after miR-337-3p overexpression (Figure 2C; Supplementary Figure S2A).

As collagen fiber disorganization is an important marker in tendinopathy, we further examined collagen alignment of patellar tendons by Sirius red staining under polarized light microscopy. The amount of collagen I fiber reduced at collagenase I group and NC group, and the orientation of collagen I was in disarray (Figure 2D; Supplementary Figure S2B). After miR-337 overexpressing, the amount and the arrangement of collagen I were significantly rescued (Figure 2D; Supplementary Figure S2B).

To further verify the therapeutic effect of miR-337-3p overexpression histologically, hematoxylin and eosin (H&E) staining and immunohistochemistry of chondro-osteogenic genes were tested. Abundant and unordered cell proliferation in patellar tendon was observed in tendinopathy group by H&E staining (Figure 2E; Supplementary Figure S2C). However, fewer proliferative cells were observed in miR-337-3p overexpressing group, and the orientation of the cells was better arranged (Figure 2E; Supplementary Figure S2C). Meanwhile, abundant expression of osteogenic gene osteopontin (OPN) and chondrogenic gene type II collagen were observed in patellar tendon slices of collagenase I and NC group by immunohistochemistry, while these two proteins were rarely expressed at miR-337-3p overexpressing group (Figure 2F; Supplementary Figure S2C). As shown in Figure 2F, compared to the negative control group, overexpressing miR-337-3p significantly reduced the expression of chondro-osteogenic markers *Spp1* and *Col2a1* in patellar tendon of tendinopathy model. All the results indicated that miR-337-3p could be a negative controller in chondro-osteogenesis of tendinopathy.

### Increased IL-1β under mechanical loading leads to miR-337-3p decrease and chondro-osteogenic differentiation of rTDSCs

**Figure 3:**
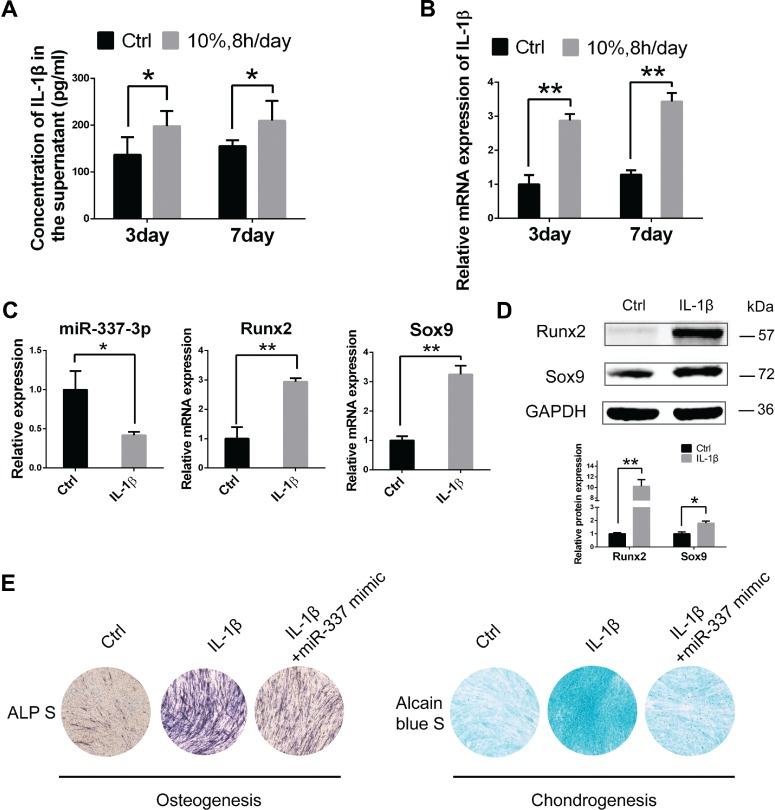
Increased IL-1β induces miR-337-3p-mediated chondro-osteogenesis of rTDSCs under mechanical loading. (**A**) Enzyme-linked immunosorbent assay detected IL-1β in the supernatant medium of rTDSCs under mechanical loading (10%, 8 h/day) or not for 3 or 7 days. (**B** and **C**) Real-time PCR analysis of *IL-1β* (**B**), rno-miR-337-3p, *Runx2*, and *Sox9* (**C**) in rTDSCs treated with 10 ng/ml IL-1β for 3 days. (**D**) Western blot analysis of Sox9 and Runx2 in rTDSCs treated with 10 ng/ml IL-1β for 3 days. The densitometric analysis of the proteins was normalized to GAPDH. Three independent experiments were analyzed for the bar graph below. (**E**) Alkaline phosphatase staining on Day 7 and Alcain blue staining on Day 14 of rTDSCs treated with IL-1β and rescued by rno-miR-337-3p mimics. Error bars, SEM (*n* = 3). *P < 0.05; **P < 0.01.

The above results have showed that miR-337-3p plays an important role in tendinopathy; however, how mechanical loading regulates miR-337-3p expression remains unsolved. It is noted that during the pathogenesis of tendinopathy, inflammation is frequently observed. We presumed that the damage to tendon caused by collagenase I treatment (Huh et al., 2009; Schelbergen et al., 2015) and mechanical loading are identical (Yang et al., 2005). Therefore, we assessed the level of IL-1β and TNF-α, two major proinflammatory cytokines, in cell culture supernatants and found that IL-1β, rather than TNF-α, was significantly increased in the 10% cyclic mechanical stretch group in rTDSCs tested on Day 3 and Day 7 (Figure 3A and B). Intriguingly, after administration of IL-1β, the expression of miR-337-3p was significantly downregulated (Figure 3C), and the level of chondro-osteogenic genes *Runx2* and *Sox9* was upregulated (Figure 3C and D). Furthermore, the ALP staining and Alcain blue staining confirmed the chondro-osteogenic differentiation of rTDSCs with IL-1β treatment, and this phenomenon was significantly rescued by miR-337-3p mimics (Figure 3E). Therefore, based on these data, we deduced that mechanical loading causes the excessive production of IL-1β, which in turn leads to miR-337-3p downregulation, and chondro-osteogenic differentiation of rTDSCs.

### IRS1 and Nox4 are direct target genes of miR-337-3p

**Figure 4:**
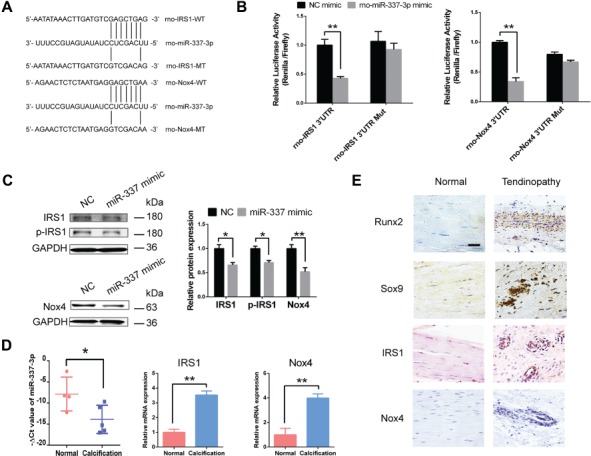
miR-337-3p targets IRS1 and Nox4 in tendinopathy. (**A**) Schematic representation of the rno-IRS1 and rno-Nox4 3′UTR indicating the binding sites of rno-miR-337-3p. WT, wild-type; MT, mutant. (**B**) HEK293T cells were transfected with psiCHECK™-2 Vector containing a fragment of rno-IRS1, rno-Nox4 3′UTR harboring binding sites for rno-miR-337-3p, or the corresponding mutant constructs. The effect of rno-miR-337 mimics on the corresponding vector luciferase activity was tested. (**C**) Protein levels of IRS1, p-IRS1, and Nox4 in rTDSCs treated with miR-337 mimics or negative control siRNAs (NC) for 3 days. The densitometric analysis of each protein expression was normalized to GAPDH. Three independent experiments were analyzed for the bar graph on the right. (**D**) Real-time PCR analysis of miR-337-3p, *IRS1*, and *Nox4* expression in normal tendon tissues obtained from four osteoarthritis patients and calcified tendon tissues from five tendinopathy patients. (**E**) Immunocytochemistry staining of Runx2, Sox9, IRS1, and Nox4 in diseased tendon of tendinopathy patients and normal tendon samples. The densitometric analysis of the proteins was normalized to GAPDH. Error bars, SEM (*n* = 3). *P < 0.05; **P < 0.01. Scale bar, 50 μm.

To figure out the downstream targets of miR-337-3p involving in chondro-osteogenic differentiation of TDSCs, we performed bioinformatic analysis using four miRNA target prediction software including TargetScan, miRanda, miRNAMap, and miRDB to screen for rno-miR-337-3p target genes. Insulin receptor substrate 1 (IRS1) and NADPH oxidase 4 (Nox4), which were previously demonstrated to be related to bone metabolism (Negishi-Koga and Takayanagi, 2012; Harrison, 2013), were chosen for further study. To test IRS1 and Nox4 were indeed the direct targets of miR-337-3p, we constructed luciferase reporter plasmids harboring the wild-type 3′UTR and the mutant 3′UTR of miR-337-3p (Figure 4A; Supplementary Figure S3A). Binding site was mutated to validate their direct targeting. Results showed that the reporter activity of the wild-type 3′UTR was significantly inhibited by miR-337-3p mimics, and this inhibition was abolished in the mutant 3′UTR (Figure 4B; Supplementary Figure S3B). Furthermore, when miR-337-3p mimics were applied, protein expression of IRS1, p-IRS1, and Nox4 were reduced in both rTDSCs (Figure 4C) and hTDSCs (Supplementary Figure S3C). All the above results demonstrated that Nox4 and IRS1 are direct targets of miR-337-3p.

Also the expression of miR-337-3p was also downregulated in the calcification tendon of tendinopathy patients, with increased IRS1 and Nox4 tested by real-time PCR (Figure 4D). Furthermore, we tested the *in situ* expression of IRS1 and Nox4 in human tendinopathy samples. The typical pathological phenomena, such as abnormal collagen arrangement and aberrant chondro-osteogenic ossification of a diseased tissue, were observed (Figure 4E). Most importantly, the expression of IRS1 and Nox4 was as predicted increased, especially in the abnormal cell proliferation zone (Figure 4E), which validated that IRS1 and Nox4 are positively related to tendinopathy.

### Mechanical loading induces chondro-osteogenic differentiation of TDSCs by increasing IRS1-dependent ERK1/2 transcription and Nox4-dependent JNK transcription

**Figure 5:**
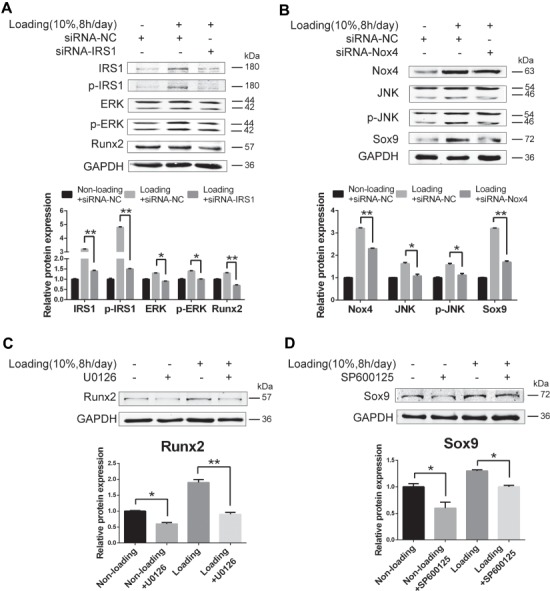
ERK1/2 pathway activated by IRS1 and JNK pathway activated by Nox4 induce chondro-osteogenic differentiation of TDSCs under mechanical loading. (**A** and **B**) rTDSCs were transfected with IRS1 silencing RNAs (**A**), Nox4 silencing RNAs (**B**), or negative control siRNA combined with mechanical loading or not for 3 days. Western blot analysis of indicated proteins was presented. (**C**) ERK pathway inhibitor U0126 was applied to rTDSCs under mechanical loading condition or not for 3 days. Protein level of Runx2 was tested. (**D**) JNK pathway inhibitor SP600125 was applied to rTDSCs under mechanical loading condition or not for 3 days. Protein level of Sox9 was tested. The densitometric analysis of the proteins was normalized to GAPDH. The lower bands of ERK, p-ERK, JNK, and p-JNK were used for densitometric analysis. Three independent experiments were analyzed for the bar graph below. *P < 0.05; **P < 0.01. Scale bar, 50 μm.

The aforementioned results have shown that protein expressions of IRS1, p-IRS1, and Nox4 were significantly upregulated in cyclic mechanical stretch group, in comparison with that in non-loading group of TDSCs (Figure 5A and B; Supplementary Figure S3D). However, how IRS1 and Nox4 regulate chondro-osteogenesis of TDSCs under mechanical loading remains obscure. In previous studies, IRS1 in osteoblast is proven to be indispensable for maintaining bone turnover through ERK and AKT signaling (Negishi-Koga and Takayanagi, 2012). Moreover, Nox4 is demonstrated to coordinate the spatiotemporal activity of JNK leading to cytoskeletal remodeling and transdifferentiation (Sampson et al., 2011).

We therefore utilized siRNAs of IRS1 and Nox4 to knock down their expression to see whether IRS1–ERK and Nox4–JNK are indeed the downstream mediators of miR-337-3p to regulate osteogenic and chondrogenic differentiation of rTDSCs, respectively. The results showed that the protein expression of IRS1, ERK, and Runx2 were downregulated after inhibition of IRS1 in rTDSCs under mechanical loading (Figure 5A). Likewise, Nox4, JNK, and Sox9 expressions decreased by Nox4 inhibition (Figure 5B). Furthermore, the protein expression of Runx2 in rTDSCs reduced when U0126 was applied to block ERK signaling, demonstrating that Runx2 is positively controlled by ERK signaling (Figure 5C). On the other hand, when SP600125 was used to block JNK signaling, similar results were obtained, indicating that Sox9 is positively controlled by JNK signaling (Figure 5D). Therefore, we concluded that IRS1 and Nox4 positively regulate the expression of Runx2 and Sox9 through ERK and JNK signaling, respectively, which leads to the chondro-osteogenesis of TDSCs under mechanical loading. In addition, both p-ERK and p-JNK levels were increased after IL-1β treatment in rTDSCs (Supplementary Figure S4), as well as under mechanical loading.

### miR-337-3p weakens chondro-osteogenic differentiation potentials of rTDSCs in collagenase I-induced rat tendinopathy model

**Figure 6:**
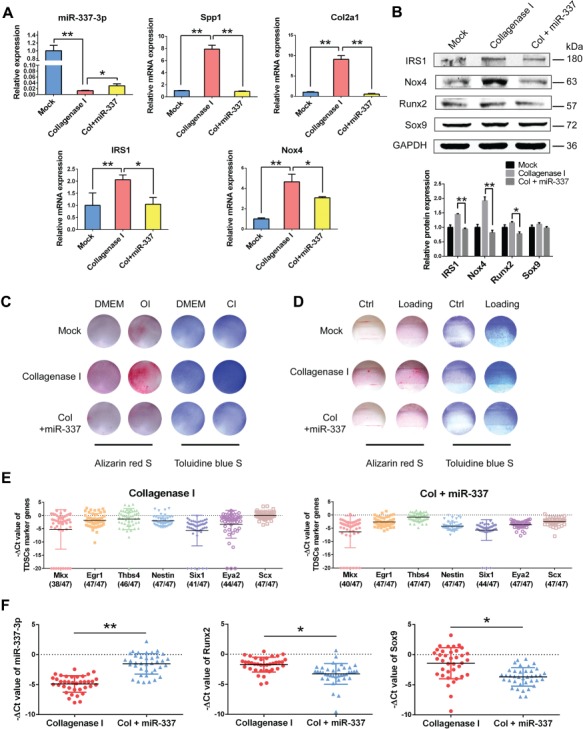
rTDSCs from rat tendinopathy group and miR-337-3p curing group present different chondro-osteogenic differentiation ability. (**A**) Real-time PCR analysis of miR-337-3p and indicated genes of rTDSCs derived from each group. (**B**) Western blot analysis of miR-337-3p target genes and chondro-osteogenic genes in rTDSCs derived from each group. The densitometric analysis of each protein expression was normalized to GAPDH. Three independent experiments were analyzed for the bar graph below. (**C**) Alizarin red staining (left) and Toluidine blue staining (right) of rTDSCs derived from each group cultured in regular medium (DMEM), osteogenic-induced medium (OI), or chondrogenic-induced medium (CI) for 10 days. (**D**) Alizarin red staining (left) and Toluidine blue staining (right) of rTDSCs derived from each group with or without mechanical loading (10%, 8 h/day) for 10 days. (**E** and **F**) Single-cell PCR analysis of flow sorted rTDSCs (CD90+, CD45−) freshly derived from collagenase I-treated group and miR-337 overexpressing lentivirus group. (**E**) The expression of TDSC marker genes *Mkx*, *Egr1*, *Thbs4*, *Nestin*, *Six1*, *Eya2*, and *Scx* was normalized to *Gapdh*. (**F**) The expression of miR-337-3p, *Runx2*, and *Sox9* in single cells with positive TDSC markers in either collagenase I-treated group or miR-337 overexpressing group. Numbers in parenthesis indicate cells positive for TDSC markers. Error bars, SEM (*n* = 3). *P < 0.05; **P < 0.01.

**Figure 7:**
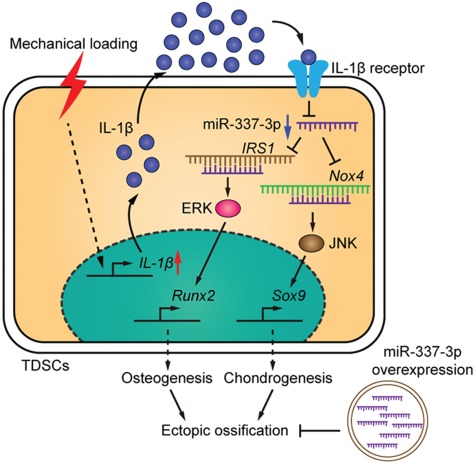
A schematic diagram shows that excessive mechanical loading-induced ectopic ossification is rescued by miR-337-3p overexpression. Downregulation of miR-337-3p by mechanical loading in TDSCs is mediated by the increase of inflammatory factor IL-1β. Nox4 and IRS1 are target genes of miR-337-3p. IRS1-activated JNK–Sox9 and Nox4-activated ERK–Runx2 pathways induce chondro-osteogenic differentiation of TDSCs, which contributes to the ectopic ossification of tendon.

Although the ectopic ossification is significantly rescued by intra-tendon injection of miR-337-3p overexpressing lentivirus in rat tendinopathy model, how miR-337-3p functioned in this curing process was not discussed. For this reason, we isolated rTDSCs from each treatment group after 12 weeks and cultured *in vitro* to further investigate whether miR-337-3p modulated rTDSCs differentiation direction by targeting IRS1 and Nox4. Expression of miR-337-3p in rTDSCs cultured *in vitro* decreased greatly in collagenase I-treated group in comparison with the mock group and was significantly rescued in miR-337 overexpressing lentivirus group (Figure 6A). As the target genes of miR-337-3p, mRNA expression of *IRS1* and *Nox4* exerted the opposite way (Figure 6A). Meanwhile mRNA expression of osteogenic gene *Spp1* and chondrogenic gene *Col2a1* was significantly rescued in rTDSCs of miR-337 overexpressing group (Figure 6A). In addition, protein level changes of IRS1, Nox4 were consistent with mRNA expression changes (Figure 6B). Runx2 and Sox9 changed slightly, which might correlate with the time courses of chondro-osteogenesis. Furthermore, the results of Alizarin red staining and Toluidine blue staining showed that rTDSCs from miR-337-3p overexpressing group presented weaker chondro-osteogenic differentiation ability compared with the tendinopathy group both in differentiation inducement incubation (Figure 6C) and mechanical loading treatment (Figure 6D). To further confirm rTDSCs differentiation was regulated by miR-337-3p that plays an essential role in tendinopathy treatment, we performed single-cell PCR to monitor the gene expression changes of TDSCs key markers (Havis et al., 2014; Lui, 2015; Yin et al., 2016) in freshly sorted TDSCs from collagenase I-treated group (negative control lentivirus) and miR-337 overexpressing lentivirus group. Results showed that these TDSCs markers, including *Mkx*, *Egr1*, *Thbs4*, *Nestin*, *Six1*, *Eya2*, and *Scx* could all be detected in most of the isolated single cells of collagenase I-treated group (37/47) and miR-337 overexpressing group (38/47) (Figure 6E). Furthermore, in these marker genes-qualified cells, expression of miR-337-3p was remarkably elevated, whereas both *Runx2* and *Sox9* levels were downregulated (Figure 6F), suggesting that miR-337 overexpressing can indeed reverse the collagenase I-induced tendinopathy symptoms via modulating rTDSCs differentiation potentials. Taken together, we deduced that miR-337-3p can be a therapeutic target of tendinopathy through downregulating chondro-osteogenic differentiation of rTDSCs by targeting IRS1 and Nox4 as elucidated in the schematic diagram (Figure 7).

## Discussion

In the present study, we for the first time identified miR-337-3p as a mechanosensitive miRNA in rTDSCs using our *in vitro* mechanical loading model and demonstrated that overexpressing miR-337-3p could effectively rescue ectopic ossification in rat tendinopathy model *in vivo*. Given that inflammation is an important external stress related to tendinopathy, we provided evidence that the proinflammatory factors IL-1β activated by mechanical loading were the upstream negative regulators of miR-337-3p in rTDSCs *in vitro*. Then we validated that IRS1 and Nox4 are direct targets of miR-337-3p in regulating chondro-osteogenesis of TDSCs. Furthermore, we demonstrated that differentiation fate of rTDSCs could be manipulated by overexpression of miR-337-3p in tendinopathy model, which contributed to the attenuation of ectopic ossification in rat tendinopathy model.

The mechanism of calcifying tendinopathy was unclear until stem cells in tendon tissues were isolated and identified (Bi et al., 2007; Rui et al., 2010). This finding provides a new angle to study basic tendon biology. In recent years, miRNAs have been proven to be important mediators in cell differentiation and emerge as a key modulator for development and pathological processes of diseases (Ambros, 2004; Kosik, 2010; Esteller, 2011; Cheng and Leung, 2018; Han et al., 2018). Until now, miR-135a was reported to modulate tendon stem cell proliferation, migration, and tenogenic differentiation via suppressing ROCK1 (Chen et al., 2015). MicroRNA29a targeting collagen III regulates IL-33-mediated collagen remodeling in human tendon after injury (Millar et al., 2015). Mechanosensitive miRNA have been found in chondrocyte (Guan et al., 2011) and human bone marrow stromal cells (Zuo et al., 2015) to regulate cell differentiation. Nonetheless, mechanosensitive miRNA that plays a role in the pathology of tendinopathy has not been reported.

Our study aimed to explore whether mechanosensitive miRNAs exist in TDSCs and play an important role in the differentiation of TDSCs that leads to chondro-ossification in tendinopathy. In this study, we for the first time identified miR-337-3p as a mechanosensitive miRNA in rTDSCs using our *in vitro* mechanical loading model. There are other more miRNAs vary between loading group and non-loading group, and thus further investigations of other functional mechanosensitive miRNAs are necessary. Several studies about miR-337-3p were focused on cancer cells (Du et al., 2012; Hauser et al., 2012; Zhang et al., 2014). miR-337-3p has been reported associated with chondrogenesis in chondrocyte by regulating transforming growth factor β receptor 2 (TGFBR2) expression (Zhong et al., 2012).

IRS1 and Nox4 were demonstrated to be the target genes of miR-337-3p for the first time in our study. We noticed that co-localization of Nox4 with the cytoskeleton and focal adhesions has been demonstrated (Hilenski et al., 2004; Clempus et al., 2007; Lyle et al., 2009), which implies Nox4 functions in the mechanosensitive signaling. Note that Nox4 in tendon disease was first analyzed in our study. IRS1 and ERK-1/2 were once demonstrated to be activated in load-induced tenocyte *in vivo* under insulin-like growth factor 1 (IGF1) signaling during the pathogenesis of overused tendon disorders although no chondro-osteogenesis was observed (Scott et al., 2007). And then IRS1 and its downstream molecules, including Akt and ERK, were found to participate in bone remodeling in IGF signaling that favors osteoblast differentiation (Negishi-Koga et al., 2011; Negishi-Koga and Takayanagi, 2012). In addition, IGF1 was reported to enhance osteogenesis of human periodontal ligament stem cells by activating ERK and JNK (Yu et al., 2012). Combing the above conclusions, we hypothesized that Nox4 and IRS1 play a role in chondro-osteogenesis of tendinopathy as target genes of miR-337-3p. In this study, we showed that Nox4 and IRS1 were positively related to chondro-osteogenic differentiation of TDSCs through JNK and ERK signaling, respectively. Our study indicated Nox4 and IRS1 as the therapeutic targets of tendinopathy; however, further research is needed to clarify the functional mechanism of Nox4 and IRS1 in chondro-osteogenesis in tendinopathy.

As the ossification of tendon is not shown in current mechanical model of tendinopathy as observed in tendinopathy patients (Huang et al., 2004; Glazebrook et al., 2008), the detection of therapeutic effects on ossification tendinopathy using this model is limited. In contrast, the phenotype of ectopic ossification in collagenase-induced tendinopathy model (Lui et al., 2009) would better resemble the real situation in athletes suffered from overuse of tendon. An excessive inflammation was showed in these two models as frequently observed in overloaded tendon (Spiesz et al., 2015; Thorpe et al., 2015). More specifically, in this study, we found that the proinflammatory cytokine IL-1β downregulates the expression of miR-337-3p; the IL-1β/miR-337-3p axis-mediated pathogenesis of tendinopathy is identical in both models. When we used collagenase-induced tendinopathy model to testify the therapeutic effect of miR-337-3p, we found increased Tenacin C and Scx expression in tendon tissue and rTDSCs (data not shown) after treatment with miR-337-3p overexpressing lentivirus. miR-337-3p may accelerate tenogenic differentiation and tendon healing via the modulation of chondro-osteogenic and tenogenic differentiation balance of rTDSCs. Whether there are tenogenic-related genes regulated directly or indirectly by miR-337-3p remains to be explored.

miRNAs are expected to be diagnostic, prognostic, therapy predictive biomarkers, and attractive therapeutic targets because of their functions in diseases (Barwari et al., 2016; Gupta et al., 2016; Biglino et al., 2017; Nassar et al., 2017; Cheng and Leung, 2018; Zhu et al., 2019). More human tendinopathy tendon and blood samples are needed to verify whether miR-337-3p could be a diagnostic and predictive biomarker. Until now, no miRNA therapy has been applied clinically because of the complexity involving hundreds of genes that one miRNA may target, as well as the influence of the miRNA family wherein the regulated miRNA may belong to, and the efficiency and safety of the delivery system (Biglino et al., 2017; Zhou and Rossi, 2017). We used a viral system for local delivery of miR-337-3p in our study to ensure high transfection efficiency. Considering the security, sustainability, and tissue-specific targeting, nanopolymers for nonviral gene delivery (Xiang et al., 2012a; Xiang et al., 2012b; Tong et al., 2013) or aptamers (Zhou and Rossi, 2017) are planned to be applied in further experiments for treatment of tendinopathy.

## Materials and methods

### Animal experiment ethics

All experiments with animals were carried according to Experimental Safety of Chinese Academy of Sciences and the Committees of Animal Ethics. All animal treatments were guided by the National Institutes of Health (NIH) guidelines. Animal suffer was made to the minimum.

### rTDSCs isolation and cell culture

rTDSCs were acquired according the previous study (Rui et al., 2010) from male Sprague Dawley (SD) rats (8-week-old, weighing 250–300 g). Patellar tendons were separated for rTDSCs isolation. rTDSCs used for all experiments were from passages 1–3.

### hTDSCs isolation and identification

hTDSCs were isolated from cruciate ligament of patients who underwent joint replacement. Adipose tissue and connective tissue were removed and the ligament was minced into ~2-mm-width, 2-mm-length pieces and digested for 4 h at 37°C with type I collagenase (3 mg/ml; Sigma-Aldrich). Following steps were the same as the rTDSCs isolation and cell culture.

### Mechanical loading application

TDSCs were plated at 6-well UniFlex™ plates (Flexcell International Corporation) as previously described (Shi et al., 2012). The uniaxial cyclic mechanical stretch was applied to the cells at 0.5 Hz sinusoidal curve, and a FX-5000 T™ Flexercell® Tension Plus™ unit (Flexcell International Corporation) was applied. Then 10% elongation was chosen and the mechanical loading was applied for 8 or 16 h per day. The max elongation of the elastic membrane where cells were cultured on was 10% when 10% loading stress was applied. The cells in stretching or in static state were incubated at 37°C and 5% CO_2_. TDSCs were collected at the end of the stress cycle for further detection.

### miRNA microarray

The miRNA microarray data of the rTDSCs with or without mechanical loading of 10% elongation for 7 days were obtained using the Agilent Rat microRNA chips (v16.0; Shanghai Biotechnology Corporation). Normalized data were further analyzed by a Student–Newman–Keuls multiple comparison test. *P* < 0.05 was considered differentially expressed.

### ALP staining

Osteogenesis of rTDSCs was tested by ALP staining after cultured with or without mechanical loading for 10 days according to the protocol of the ALP staining kit C3206 (Beyotime). Photographs were obtained by the digital camera (Nikon D90) or microscope (Nikon TE2000).

### Animal model of tendinopathy

Male 8-week-old SD rats were used in this experiment. Rats were anaesthetized using 2.5% pentobarbital sodium (0.25 ml/100 g body weight). Collagenase I-induced tendinopathy rat model was built following by the process described by Chen et al. (2004). Bacterial collagenase I (20 μl of 0.015 mg/ml in saline; Sigma-Aldrich) or saline was injected into the patellar tendon intratendinously with a 29-gauge needle in one limb. Three days later, 50 μl rno-miR-337-3p-overexpressing lentivirus (1 × 10^9^ TU/ml) or negative control lentivirus (GenePharma) was injected into the patellar tendon where collagenase I treated before. After 8, 12, and 16 weeks, rats from each group were revaluated by X-ray and then sacrificed to harvest patellar tendons or rTDSCs for further detection.

### Histology and immunohistochemistry

Rat patellar tendons were fixed with 4% paraformaldehyde dissolved in PBS for 24 h. After washing with water overnight, the samples were embedded within paraffin followed by gradient alcohol dehydration and cut longitudinally to 5 μm. H&E staining was carried for analysis of the pathologic changes. Anti-osteopontin (ab8448, Abcam) and anti-type II collagen (ab34712, Abcam) were used for immunohistochemistry staining.

### Luciferase reporter assay

HEK293T cells were co-transfected with recombinant plasmid and miR-337-3p mimics or negative control siRNAs (GenePharma). Lipofectamine 2000 (Invitrogen) was used for transient transfection according to the manufacturer’s instructions. After 24 h, cells were lysed and Firefly and Renilla luciferase activities were measured by Dual-Luciferase Reporter Assay System (Promega), repeated for three times.

### Chondro-osteogenic differentiation induction of rTDSCs

Osteogenic-induced medium (OI) containing 50 μM ascorbic acid, 10 mM β-glycerophosphate, and 100 nM dexamethasone (all from Sigma-Aldrich) was applied to induce osteogenic differentiation of rTDSCs. Chondrogenic-induced medium (CI) containing 10 ng/ml transforming growth factor-β3, 50 μg/ml ascorbate-2-phosphate, 40 μg/ml proline, 50 mg/ml ITS (Invitrogen), 100 μg/ml pyruvate, and 100 nM dexamethasone was applied to induce chondrogenic differentiation of rTDSCs.

### Single-cell PCR

Freshly flow sorted CD90-positive and CD45-negative rTDSCs obtained from patellar tendon of collagenase I treatment and miR-337-3p overexpressing groups were used for single-cell PCR. Single-cell capture and cDNA preamplification were carried on the C1™ Single-Cell Preamp IFC (10–17 μm) by the C1 system (Fluidigm) as described by Yin et al. (2016). Single-cell PCR was performed with 96.96 Dynamic Array™ IFC for Gene Expression chip on the BioMark system (Fluidigm) according to manufacturer’s specifications. Cycle threshold value of *Gapdh* was used for normalization. Each gene was detected for seven times. The gene expressions of single cells were performed using GraphPad Prism 6.

### Statistical analysis

All results are presented as mean ± SD. Statistical significance of two or more groups were performed by analysis of variance (ANOVA) followed by *post hoc* test. All data analyses were carried out using SPSS software. *P* < 0.05 was considered statistically significant.

### Data availability statement

Raw data of miRNA microarray are available in the Gene Expression Omnibus (accession number GSE114828; http://www.ncbi.nlm.nih.gov/geo/query/acc.cgi?acc=GSE114828).

More details are provided in Supplementary materials and methods.

## Supplementary Material

Supplementary_material_mjz030

## Notes

**Author contributions:** conception and design by Y.G. and Xiaoling Zhang; acquisition of data by Y.G., X.Zhao, G.H., and S.-C.F.; analysis and interpretation of data by Y.G. and Xudong Zhang; writing, review, and revision of the manuscript by Y.G. and Xiaoling Zhang; technical or material support by J.X., X.C., and Y.S.-H.P.; study supervision by K.D. and Xiaoling Zhang.
