# Supplementary material for: Overexpression of mechanical sensitive miR-337-3p alleviates ectopic ossification in rat tendinopathy model via targeting IRS1 and Nox4 of tendon-derived stem cells
Source: J Mol Cell Biol. 2019 May 8;12(4):305–17. doi: 10.1093/jmcb/mjz030 (PMC7232128; doi:10.1093/jmcb/mjz030)
Supplement: Supplementary_material_mjz030 [file supplementary_material_mjz030.pdf]

Supplementary Figures

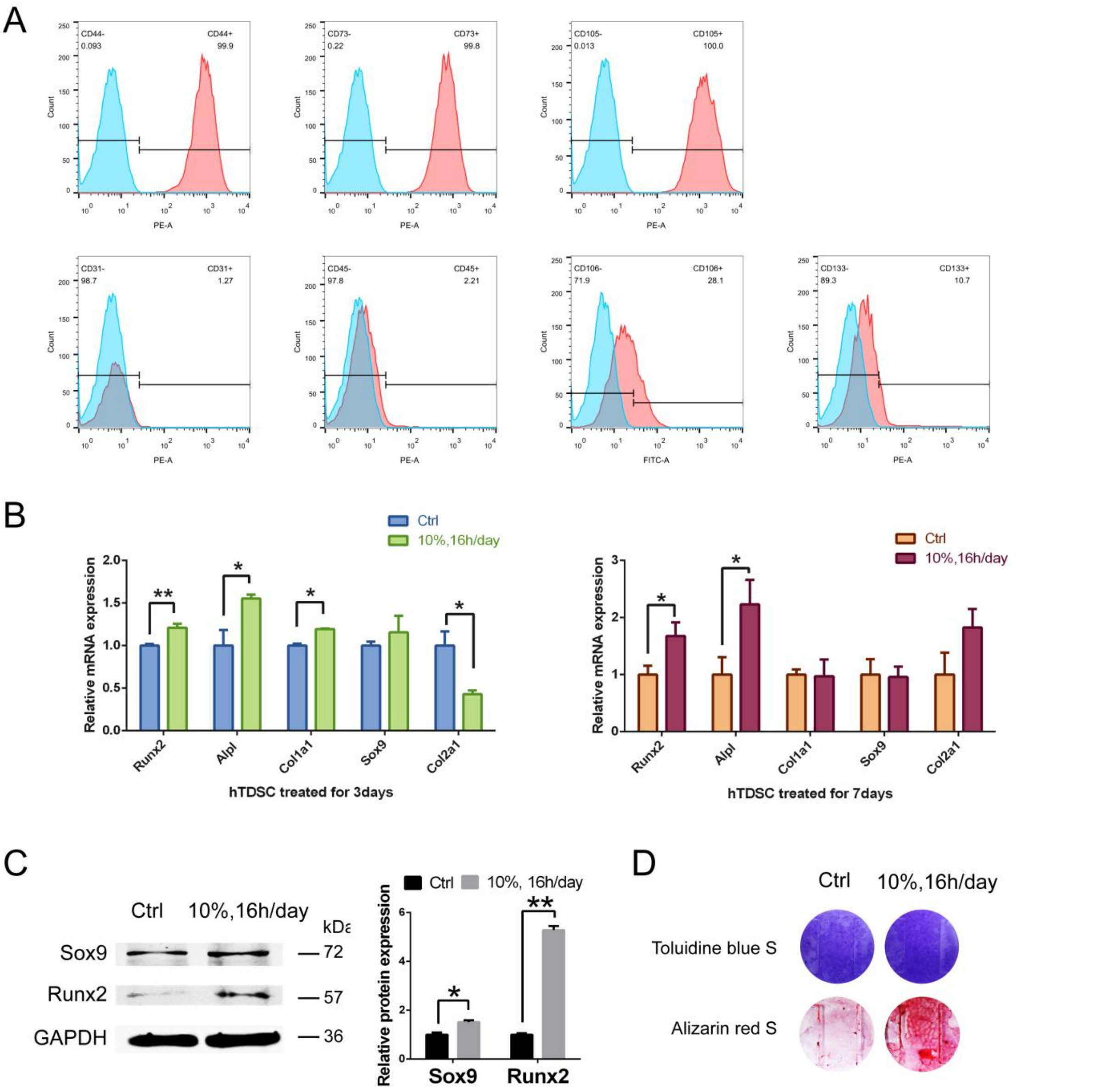

Supplementary Figure S1. hTDSCs turn into osteogenetic differentiation with decreased miR-337-3p under mechanical loading.

(A) Flow cytometry analysis of the expression of positive (above) and negative(below) cell surface markers to hTDCSs. (B) Real-time PCR analysis of chondro-osteogenic genes in hTDSCs with or without mechanical loading (10%, 16h/day) at day 3(above) and day 7(below). (C) Protein levels of Sox9 and Runx2 in hTDSCs with or without mechanical loading (10%, 16h/day) for 7days. The densitometric analysis of three protein expression levels was normalized to GAPDH. Three independent experiments were analyzed for the bar on the right. (D) Toluidine blue staining (above) and alizarin red staining (below) of hTDSCs with or without mechanical loading (10%, 16h/day) for 14days. Error bars, SEM (n=3). *P < 0.05; **P < 0.01.

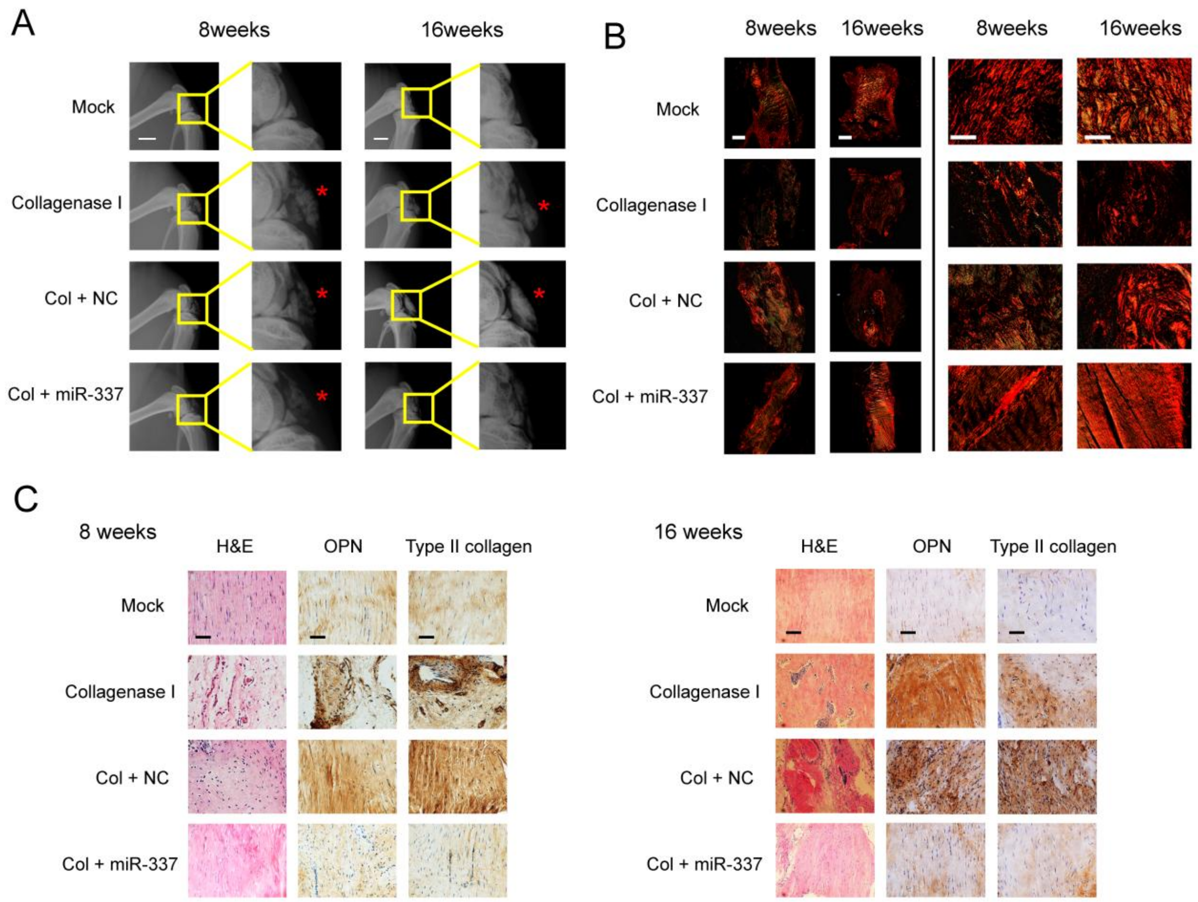

Supplementary Figure S2. miR-337 overexpressing lentivirus treat rat tendinopathy model for 8 weeks or 16 weeks.

(A) X-ray image of the knees of SD rats at 8 weeks and 16weeks after treatment showed knee joints and ectopic ossicles (red asterisks in the magnified pictures) formed in patellar tendons (left). Bars, 5 mm. (B) Patellar tendon paraffin sections collected from each group treated with sirius red staining observed under polarized light microscopy to observe the collagen fibers. Left column were integrated intact patellar tendon images. Right column were enlarged partial patellar tendon images. Bars (left), 800 μm. Bars (right), 200 μm. (C) H&E staining and immunocytochemistry staining of chondro-osteogenic genes, tenogenic genes and miR-337-3p target genes in each group. Samples were collected 8 weeks (left) or 16 weeks (right) after treatment. Bars, 50 μm

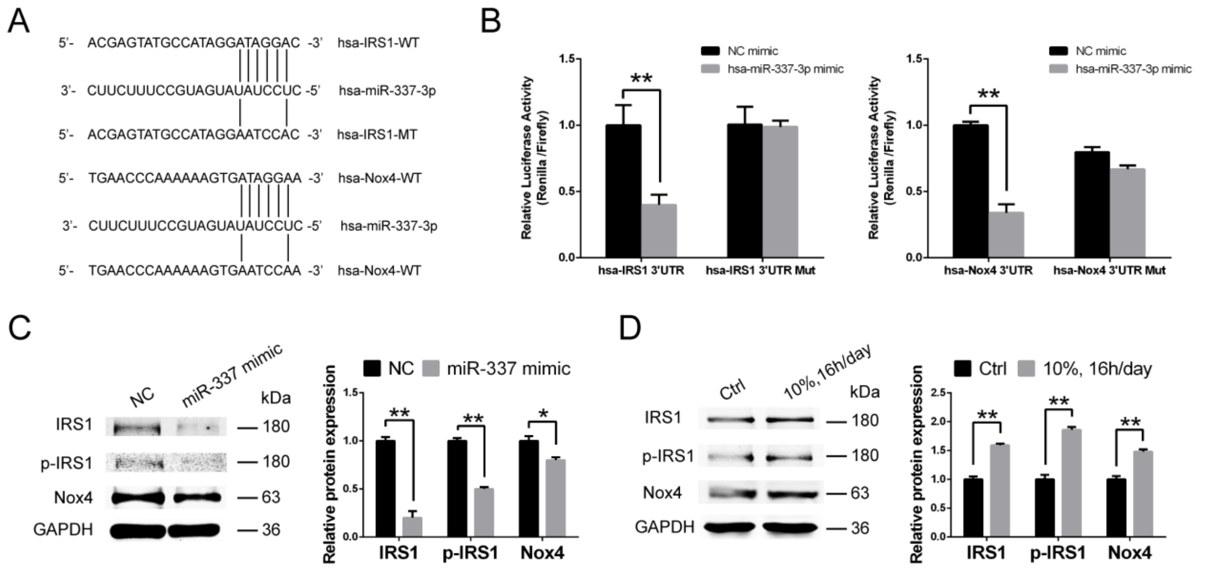

Supplementary Figure S3. hsa-miR-337-3p targets IRS1 and Nox4 in hTDSCs

(A) Schematic representation of the hsa-IRS1 and hsa-Nox4 3'UTR indicating the binding sites of hsa-miR-337-3p. WT, wildtype; MT, mutant. (B) HEK293T cells were transfected with either psiCHECK™-2 Vector containing a fragment of hsa-IRS1 or hsa-Nox4 3'UTR harboring binding sites for hsa-miR-337-3p, or the corresponding mutant constructs. The effect of hsa-miR-337 mimics on the corresponding vector luciferase activity was tested. (C) Protein levels of IRS1, p-IRS1 and Nox4 in hTDSCs treated with miR-337 mimics or negative control siRNAs (NC) for 3days. (D) The protein level of IRS1, p-IRS1 and Nox4 in hTDSCs with or without mechanical loading (10%, 16h/day) for 3days. The densitometric analysis of the proteins was normalized to GAPDH. Three independent experiments were analyzed for the bar graph on the right. Error bars, SEM (n=3). *P < 0.05; **P < 0.01.

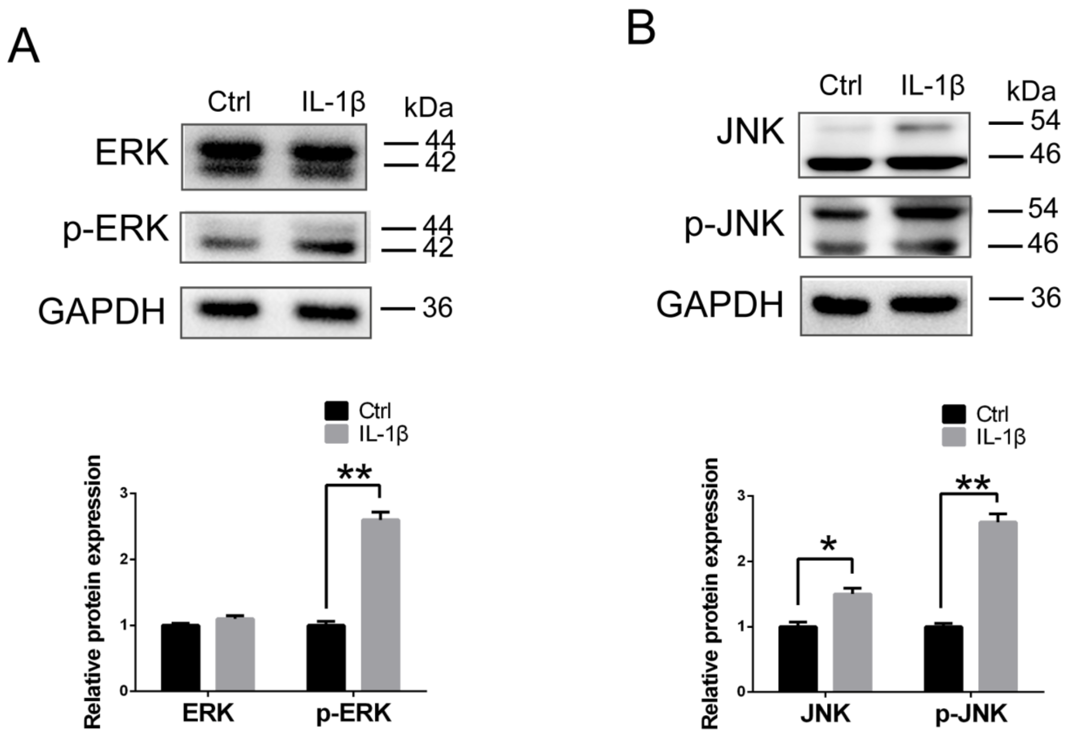

Supplementary Figure S4. ERK and JNK pathways were activated under IL-1β treatment of rTDSCs

Western blot analysis of ERK, p-ERK (A) and JNK, p-JNK (B) in rTDSCs treated with 10ng/ml IL-1β for 2days.The densitometric analysis of the proteins was normalized to GAPDH. The lower bands of ERK, p-ERK, JNK and p-JNK were used for densitometric analysis. Three independent experiments were analyzed for the bar graph below. *$P < 0.05$; **$P < 0.01$.

Supplementary materials and methods

Real-time PCR

The procedure was carried according to the previous protocol (Shi et al., 2007). *Gapdh* was applied as the internal control. The primer sequences used in this study were as follows: *Gapdh* (Glyceraldehyde-3-phosphate dehydrogenase) (accession no. NM_017008.3) forward 5'-CTCAACTACATGGTCTACATGTTCCA-3' and reverse 5'-CTTCCCATTCTCAGCCTTGACT-3'; *Runx2* (accession no. NM_053470.1) forward 5'-CGGAGCGGACGAGGCAAGAG-3' and reverse 5'-AGAGTCATCAAGCTTCTGTCTGTGC-3'; *Alpl* (accession no.NM_013059.1) forward 5'-CGTCTCCATGGTGGATTATGC-3' and reverse 5'-TGGCAAAGACCGCCACAT-3'; *Col1a1* (accession no. NM_053304.1) forward 5'-TTCACCTACAGCACGCTTGTG-3' and reverse5'-GATGACTGTCTTGCCCCAAGTT-3'; *Spp1* (accession no. NM_012881) forward 5'-CCAAGCGTGGAAACACACAGCC-3' and reverse 5'-GGCTTTGGAACTCGCCTGACTG-3'; *Sox9* (accession no. NM_080403) forward 5'-CGTCAACGGCTCCAGCA-3' and reverse 5'-TGCGCCCACACCATGA-3'; *Col2a1* (accession no. NM_012929.1) forward 5'-CCGGACTGTGAGGTTAGGAT-3' and reverse 5'-AACCCAAAGGACCCAAATAC-3'; *IL-1β* (accession no. NM_031512.2) forward 5'-CCTTGTGCAAGTGTCTGAAGC-3' and reverse 5'-CCCAAGTCAAGGGCTTGGAA-3'; *IRS1* (accession no. NM_012969.1) forward 5'-GCAACCGCAAAGGAAATG-3' and reverse 5'-ACCACCGCTCTCAACAGG-3'; *Nox4* (accession no. NM_ 053524.1) forward 5'-TTCTCAGGTGTGCATGTAGC-3' and reverse 5'-CGGAACAGTTGTGAAGAGAAGC-3'.

Western blot analysis

The procedure was carried according to the previous protocol (Shi et al., 2007). The primary antibodies used were anti-Runx2 (mouse monoclonal to Runx2) (ab54868, Abcam, USA) at a dilution of 1:1000; anti-Sox9 (rabbit polyclonal to Sox9) (ab26414, Abcam, USA) at a dilution of 1:1000; anti-IRS-1 (mouse monoclonal to IRS-1) (#3194,Cell signaling, USA), anti-p-IRS-1 (rabbit polyclonal to phosphor-IRS-1) (#3203,Cell signaling, USA), anti-Nox4 (monoclonal to NADPH oxidase 4) (ab109225, Abcam, USA). Anti-GAPDH antibody

(mouse monoclonal GAPDH antibody) (CW0100, CWBIO, China) was used at a dilution of 1:5000 for the normalization.

Construction of 3'UTR luciferase reporter plasmids and the mutants

The recombinant plasmids were created by connecting the fragments harboring the 3' UTR of IRS1or Nox4 to the sites, Xhol and NotI of the psiCHECK™-2 Vector (Promega, USA). The following primers were used for clone of 3' UTR. rno-IRS1: sense, 5'-CCGCTCGAGAATGTAACTTTCCTCACAGCAC-3'; and antisense, 5'-ATAAGAATGCGGCCGCTCCAATTCTAAGCCGACAC-3'. rno-Nox4: sense, 5'-CCGCTCGAGCTACTGGGACTCTAAAGAAGGA-3'; and antisense, 5'-ATAAGAATGCGGCCGCAGAGGGCTGTGGCTATCA-3'. hsa-IRS1: sense, 5'-CCGCTCGAGTGTAGCCTGTTGTGTGTCTCCC-3'; and antisense, 5'-ATAAGAATGCGGCCGCCCTACAAATGTCTATGCAA-3'. hsa-Nox4: sense, 5'-CCGCTCGAGGCAGTGTGACTTACCTTGCCAT-3'; and antisense, 5'-ATAAGAATGCGGCCGCGACTGTTGTCAAGACATAC-3'. The mutated 3' UTR plasmids were produced by site-directed mutagenesis using the QuikChange lightning site-directed mutagenesis kit (Stratagene, USA). The following primers were used. rno-IRS1: sense, 5'-AATATAAACTTGATGTCGTCGACAGTTCCTTTTAAGAATTAA-3'; and antisense, 5'-TTAATTCTTAAAAGGAACTGTCGACGACATCAAGTTTATATT-3'. rno-Nox4: sense, 5'-GAACTCTCTAATGAGGTCGACAACTCACTCACTCTGA-3'; and antisense, 5'-TCAGAGTGAGTGAGTTGTCGACCTCATTAGAGAGTTC-3'. hsa-IRS1: sense, 5'-ACGAGTATGCCATAGGAATCCACAAATTCAGTAAACAGGA-3'; and antisense, 5'-TCCTGTTTACTGAATTTGTGGATTCCTATGGCATACTCGT-3'. hsa-Nox4: sense, 5'-TGAACCCAAAAAAGTGAATCCAAATGTGGAATGCTCATGC-3'; and antisense, 5'-GCATGAGCATTCCACATTTGGATTCACTTTTTTGGGTTCA-3'.All sequences of the amplified products were confirmed by DNA sequencing.

IRS1 and Nox4 knockdown by siRNA

Interference of mRNA was performed using siRNA (GenePharma, Shanghai, China) designed targeting rat IRS1 or Nox4. Transfection for rTDSCs was carried out using siRNA-Mate™

reagent (GenePharma, China) according to the manufacturer’s protocol. The efficiency of transfection was assessed by FAM labeled siRNA (GenePharma, China).
